# The role of UGT1A1 polymorphism in the management of colorectal cancer

**DOI:** 10.3389/or.2025.1547904

**Published:** 2025-05-13

**Authors:** Elham Babadi, Kamran Roudini, Kianmehr Aalipour, Olatunji B. Alese

**Affiliations:** ^1^ Department of Hematology/Medical Oncology, Winship Cancer Institute of Emory University, Atlanta, GA, United States; ^2^ Department of Hematology/Medical Oncology, Tehran University of Medical Sciences, Tehran, Iran; ^3^ Digestive Disease Research Institute, Tehran University of Medical Sciences, Tehran, Iran

**Keywords:** colorectal cancer, UGT1A, irinotecan, population, pharmacogenenomics and personalised medicine

## Abstract

Colorectal cancer is a leading cause of cancer related deaths among patients worldwide, necessitating the development of more effective and tolerable therapies. Topoisomerase I inhibitors such as Irinotecan are integral components of chemotherapy regimens used in the management of colorectal, as well as esophageal, gastric, biliary tract, pancreatic, neuroendocrine, small bowel and anal carcinomas. Efficacy and toxicity of these regimens are however impacted by metabolism via the UGT1A1 pathways. This literature review provides a comprehensive overview of UGT1A1 polymorphism in patients with colorectal cancer, including recent developments and the future landscape. Recent literature elucidating the roles of oncogenes and predictive biomarkers on anti-cancer drugs and UGT1A1 genotypes are described. The lack of consensus in the clinical management of patients with colorectal cancer were also explored in depth. A comprehensive search was performed in multiple databases (including PubMed, Embase, Web of Science, Scopus, Research gate, and Google Scholar) to identify relevant articles published up to January 2024. A total of 79 clinical studies were included in this review. The epidemiology and frequency of UGT1A1 genes polymorphisms by race, gender, ethnicity, geographic location and stage of the cancer were correlated with drug metabolism, toxicity, and survival outcomes. The tole of UGT1A1 as a prognostic and predictive biomarker, including existing challenges in clinical application were also discussed extensively.

## Introduction

Colorectal cancer (CRC) is the third most commonly diagnosed cancer worldwide, accounting for approximately 10% of all cancer cases. CRC is also the second leading cause of cancer-related deaths across the world. It primarily affects elderly people, especially individuals aged 50 and above. However, it is poised to become the leading cause of cancer-related deaths in individuals under 50 regardless of gender (1, 2, 3). Sedentary lifestyle, obesity, excessive alcohol consumption and smoking, and unhealthy diet contribute to CRC development. Despite numerous studies establishing the role of screening methods such as colonoscopy and stool DNA tests, CRC is often diagnosed at advanced stages when treatment options are limited (4, 5, 6). In recent years, many biomarkers have been described for colorectal cancer management (7, 8). Irinotecan (IRI) is a chemotherapy agent used in multiple gastrointestinal (GI) malignancies, including CRC. It is associated with improved outcomes, and it prolongs survival in advanced CRC. Response varies between individuals but predictive biomarkers such as coding and non-coding variants related to drug response and adverse events are poorly understood (9, 10, 11, 12, 13).

Enzymes known as UDP-glucuronosyltransferases (UGTs) play a crucial role in IRI metabolism. These enzymes convert IRI’s active form (SN38) into SN38 glucuronide (SN38G). The genes translated into UGT enzymes belong to the UGT gene family, which includes various UGT1As. Importantly, UGT1A1 is crucial for the glucuronidation process, and variations in the UGT1A1 gene are significantly linked to IRI metabolism (14, 15, 16, 17). To date, a wide range of studies have been conducted on how IRI affects colorectal cancer patients with various UGT1A1 genotypes but controversies exists regarding a clear pathway (18, 19, 20, 21). This literature review aims to provide a comprehensive overview of the impact of UGT1A1 polymorphism in patients with CRC. A comprehensive database search was performed, including PubMed, Embase, Web of Science, Scopus, and Google Scholar to identify relevant articles published up to January 2024. All clinical-based studies reporting the UGT1A1 polymorphism in patients with colorectal cancer were identified and included in this review.

Neutropenia is a significant side effect of irinotecan (IRI) in many chemotherapy regimens used in colorectal cancer treatment. It is commonly a dose limiting toxicity for patients receiving FOLFIRI, FOLFOXIRI and many variations of Irinotecan given in combination with biologics such as Vascular endothelial growth factor (VEGF) or Epidermal growth factor receptor (EGFR) inhibitors. Specific gene polymorphisms, especially UGT1A16 and 28, can help predict this toxicity. A meta-analysis of 12 studies involving 746 cases with the wild-type genotype (G/G) and 394 cases with variant genotypes (G/A and A/A) revealed that individuals with UGT1A16 polymorphisms face a much higher risk of developing severe neutropenia, particularly in Asian populations (such as those in China and Japan). These findings highlight UGT1A16 polymorphisms as key risk factors for IRI-induced neutropenia in certain cancer patients (22).

### UGT enzymes and metabolism of irinotecan

UGT1 and UGT2 are two subfamilies and 35 products of the *UGT* gene. *UGTs* have been described in extrahepatic tissues like the brain, kidney, gastrointestinal tract apart from the liver (23). UDP-glucuronosyltransferases are enzyme products of UGT1 gene translation in some mammals like humans and rodents. UGT1 products have been implicated in carcinogenesis, mutagenesis, and drug toxicities. They are related to specific exons with specified promoter elements and help in bilirubin metabolization. Crigler-Najjar type 1 is a metabolic disease related to UGT1A1 mutation in humans. The severity of the disease has direct relation with enzyme function, which is in turn related to the amount of repeats of TA in TATA box of proximal part of UFT1A1 (24).

The UGT1 complex locus, initially believed to contain six UDP-glucuronosyltransferase genes, encodes 13 isoforms, designated *UGT1A1* to *UGT1A13p*. Among these, *UGT1A2p*, and *UGT1A11p* to *UGT1A13p* are classified as pseudogenes due to mutations that render them nonfunctional. The locus features 13 distinct exons, each preceded by a TATA box, linked to four shared exons. This enables separate transcription initiation and the production of overlapping transcripts. Of the nine functional transcripts, each begins with a unique exon that determines substrate specificity, while the shared exons encode a common region that binds UDP-glucuronic acid (25) Notably, UGT1A1 plays a vital role in bilirubin metabolism. The first exons are organized into two groups: cluster A (UGT1A2p to UGT1A5) and cluster B (UGT1A7 to UGT1A13p). UGT1A1 behaves more like cluster A and UGT1A6 resembles the other cluster. The locus has recently been expanded from 95 kb to 218 kb due to the identification of additional exons. The mRNA variants from this locus exhibit tissue-specific expression patterns, illustrating the efficient use of a limited number of exons to generate a diverse array of transferase enzymes capable of metabolizing a broad spectrum of substrates (25).

**FIGURE 1:**
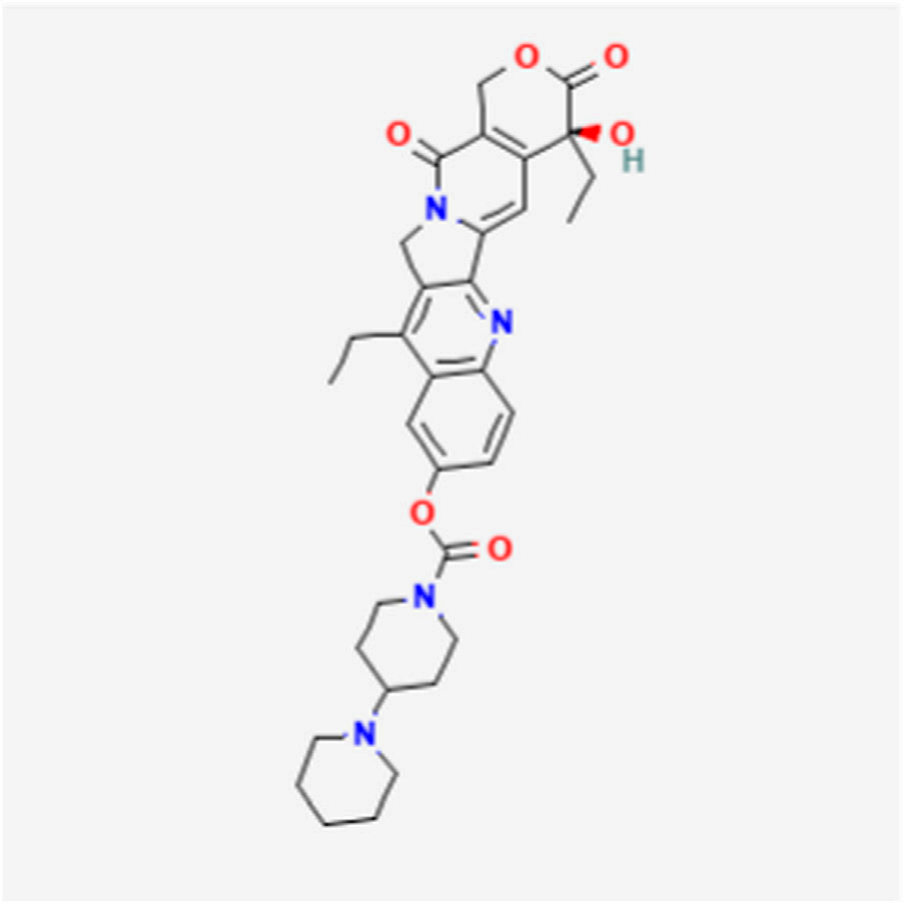
Irinotecan chemical structure.

**FIGURE 2:**
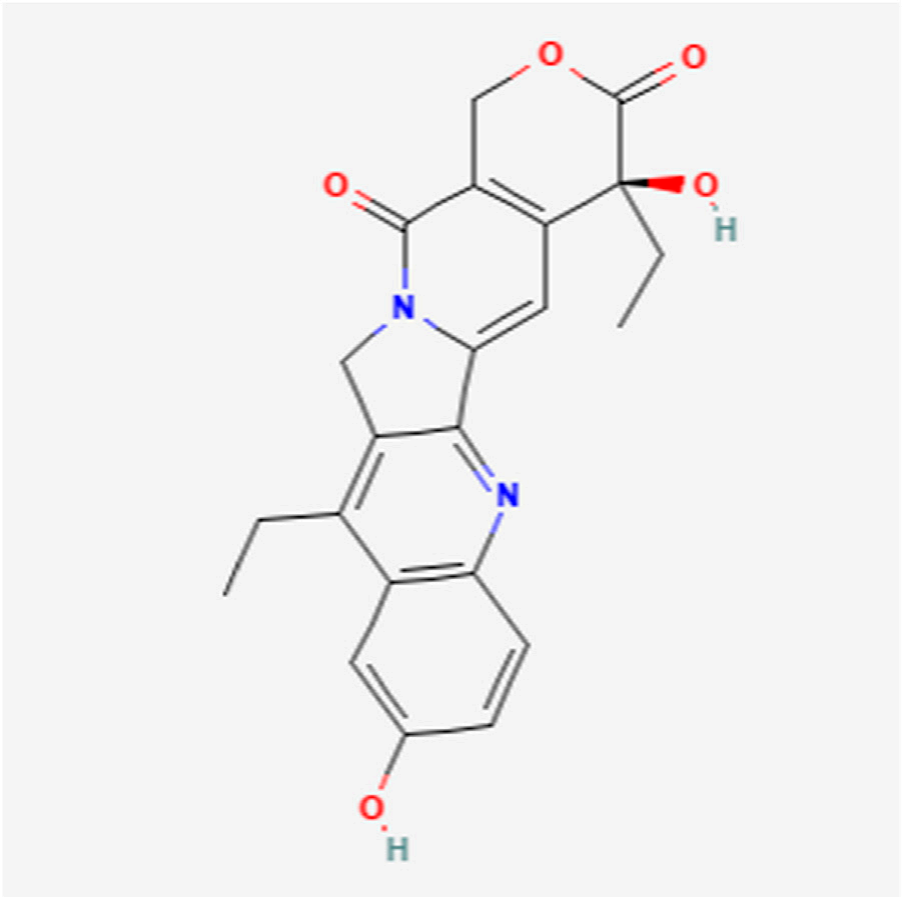
SN-38 chemical structure.

UGT1A1 and UGT1A9 enzymes metabolize SN-38, the active form of the cancer drug irinotecan (Figures 1, 2) (26, 27). Variations in SN-38 metabolism have been associated with specific genetic variants in Asian and Caucasian populations. Some UGT1A9 alleles were more common in Asians, while others were rare or absent in Caucasians, and vice versa (28). Individual and tissue-specific variations in UGT1A enzyme distribution and expression are linked to disease susceptibility and differences in substance metabolism. The role of epigenetics, such as DNA methylation and histone modification, gene expression regulation at the transcriptional level, and microRNAs (miRNAs) in posttranscriptional control are being explored. Epigenetics influence the regulation of the UGT1A enzyme family and its role in UGT1A-related diseases and therapies, offering guidance for future research endeavors (29). Studies reveal that cells lacking UGT1A1 show abnormal methylation at specific CpG sites, inversely affecting gene expression. One such site, CpG-4, is near elements that activate UGT1A1 expression and is also a binding site for regulatory proteins. Mutations in UGT1A1 can cause disorders like Gilbert syndrome and Crigler-Najjar syndrome. DNA methylation of HNF1A may significantly regulate drug metabolism and transporter pathways, affecting the local inactivation of drugs like the anticancer agent SN-38 through glucuronidation and influencing the tumor’s response to treatment (14, 30).

Certain variant alleles that are less effective at detoxifying carcinogens are associated with a higher risk of colorectal cancer and hepatocellular carcinoma (31). Pharmacogenomics has shown that genetic variations in UGT1A1 and ABCC2 influence the metabolism of Irinotecan and its associated side effects. While genotyping these genes can help predict adverse reactions, the protective mechanisms against severe side effects, such as diarrhea, remain unclear (32). The likelihood of hematologic toxicity from Irinotecan is related to the drug dosage and also the presence of the UGT1A1-28 genotypes (33). Mutations in UGT1A1-6 and UGT1A1-28 are associated with increased risk and severity of side effects in patients treated with IRI (CPT-11) for various cancers. Specifically, the UGT1A1-6 mutation is linked to a higher incidence of diarrhea in CRC patients. However, genotype variations and dosage adjustments do not appear to affect the overall effectiveness or prognosis of the treatment in other populations (34).

### The role of UGT1A1 in colorectal cancer

CRC remains a significant health concern, with disparities in incidence and mortality rates among various racial and ethnic groups. It is one of the leading causes of cancer-related deaths in the United States. It is estimated that 152,810 individuals will be diagnosed with CRC in 2024 alone, leading to 53,010 deaths (1).

#### The global variation in a genetic biomarker and its association with CRC

Recent advances in the understanding of the molecular mechanisms of colorectal cancer (CRC) have improved the selection of management strategies. Biomarkers such as microsatellite instability (MSI), or mutations in the *BRAF, KRAS*, and *HER2* genes are increasingly used in clinical practice to select chemotherapy and newer targeted therapies (35), including monoclonal antibody therapies like cetuximab and panitumumab, which target the epidermal growth factor receptor (36). Most polymorphisms have not been validated as predictive biomarkers and are, therefore, not currently suitable for clinical use. A meta-analysis of 386 potentially relevant studies revealed that the UGT1A1-6 polymorphism is associated with adverse reactions caused by Irinotecan in CRC. This association is particularly notable for the increased incidence of severe late-onset diarrhea and neutropenia. However, no correlation was observed between the UGT1A1 polymorphism and therapeutic response (6). Genotyping UGT1A1 alleles is often recommended before initiating Irinotecan treatment to prevent severe adverse effects (35).

An evidence-based review found the strongest association with severe neutropenia. Patients who are homozygous for the UGT1A1-28 allele are 3.5 times more likely to experience severe neutropenia compared to those with the standard genotype. The clinical value of UGT1A1 genotyping lies in reducing drug-related adverse reactions while preserving tumor response rates and minimizing morbidity/mortality. Strategies to mitigate risk include adjusting the Irinotecan regimen, using alternative drugs, or administering colony-stimulating factors. However, prospective studies examining these approaches are lacking, and further research is needed (9). A separate study examined the influence of various genetic variants within the UGT1A genes on the severity of side effects and the efficacy of the combination of 5-Fluorouracil (5FU) and Irinotecan chemotherapy (FOLFIRI). In addition to UGT1A1-28, the study included UGT1A1-60, UGT1A1-93, UGT1A73, and UGT1A9-22. It involved 250 patients with mCRC, analyzing the relationship between these genetic markers, hematologic and non-hematologic side effects, treatment response, disease progression, and overall survival. The results suggest that genotyping multiple UGT1A variants may significantly improve the prediction of outcomes for patients undergoing FOLFIRI (37).

In another study of 67 CRC patients treated with Capecitabine and Irinotecan, UGT1A7 and UGT1A9 genotypes were potential predictors of treatment response and toxicity. Specifically, patients with reduced UGT1A7 activity or the UGT1A9 (dT)9/9 genotype showed a stronger antitumor response with fewer adverse effects (38). Furthermore, an investigation into how 16 INDEL polymorphisms are associated with CRC risk and clinical characteristics in a mixed population included 140 CRC patients and 140 controls, with genomic DNA extracted from blood samples. Polymorphisms and genomic ancestry were analyzed using Multiplex-PCR and capillary electrophoresis. Clinical data were collected from patient records. Logistic regression analysis revealed that IL4 gene variations increased CRC risk, while TYMS and UCP2 were associated with decreased risk. Specific INDELs directly correlated with tumor location, metastasis risk, relapse, and ten-year mortality. Variations in *ACE, UCP2, TYMS, IL4, NFKB1, CASP8, TP53, HLAG, UGT1A1,* and *SGSM3* were linked to CRC risk and clinical characteristics, suggesting that this genetic marker panel could enhance clinical management. However, validation through prospective trials is necessary for adoption (39).

#### UGT1A1 polymorphism in Japanese population

A study examined the influence of UGT1A1 genetic polymorphisms on drug toxicity in 199 Japanese patients undergoing 5FU, Irinotecan and Oxaliplatin (FOLFIRINOX) chemotherapy. For patients who received a modified FOLFIRINOX regimen, there were no notable differences in the frequency of adverse events based on UGT1A1 status. However, patients with heterozygous UGT1A1 polymorphisms who were treated with the standard FOLFIRINOX regimen experienced severe toxicity more frequently than patients with wild-type UGT1A1 (WT) (40). In another study, 177 Japanese cancer patients were treated with Irinotecan, either as monotherapy or in combination with other chemotherapy agents. The researchers analyzed the diplotypes of UGT1A gene segments, specifically UGT1A1, UGT1A7, UGT1A9, UGT1A10, and Block C which includes common exons 2–5. They also evaluated the combined haplotypes of UGT1A9-1A7-1A1. The aim of study was to determine the relationship between these diplotypes and the incidence of adverse effects in 55 patients who received Irinotecan monotherapy. The findings suggested that haplotypes associated with decreased area under the curve (AUC) ratios and an increased risk of neutropenia included UGT1A1-6 or UGT1A1-28. Consequently, genotyping both variants is recommended prior to administering Irinotecan to Japanese patients, and possibly to other Asian populations as well. Additionally, the study revealed that patients with haplotypes containing UGT1A1-6 or UGT1A1-28 showed a significant decrease in their AUC ratios. The effects of UGT1A1-6 and UGT1A1-28 were similar in magnitude. In a multivariate analysis, individuals homozygous or double heterozygous for *6 and 28 (UGT1A16/*6, *28/*28, and *6/*28) were significantly associated with severe neutropenia among the 53 patients who received Irinotecan as a single agent (41).

In a retrospective study of 42 consecutive Japanese patients with advanced colorectal cancer between April 2005 and December 2009 at Saitama Medical University Hospital and International Medical Center, genotyping revealed UGT1A1 in 24 patients, and UGT1A1-6/UGT1A1-28 in 18 patients. The study aimed to compare the efficacy and toxicity of FOLFIRI as first-line chemotherapy in patients with the UGT1A1 genotype compared with the UGT1A1 (*1/*6 or 1/28) genotype. The study reported no difference in the efficacy and side effects of FOLFIRI among the different UGT1A1 genotypes (such as UGT1A1, UGT1A1-6, and UGT1A1-28). Patients with different genotypes could therefore receive the same therapy (42). In a study of 84 Japanese cancer patients, comprising 50 with colon cancer, 18 with stomach cancer, seven with ovarian cancer, seven with lung cancer, and two with other types of cancer, a genetic association between UGT1A7 and UGT1A9 polymorphisms and the UGT1A1-6 allele was reported. This genetic association correlated with reduced activity of SN-38. This is attributed to the lower catalytic and transcriptional functions of UGT enzymes, affecting Irinotecan’s metabolism and leading to decreased glucuronosyltransferase activity for SN-38 (43).

The 2010 guidelines from the Japanese Society for Cancer of the Colon and Rectum (JSCCR) address the treatment of colorectal cancer and emphasize the importance of understanding the role of the UGT1A1 gene in metabolizing the active form of SN-38 to its inactive form, SN-38 G. It highlights individuals with specific UGT1A1 genetic variations—such as double heterozygotes for *6 and *28, or homozygotes for *6 or *28, are at risk of delayed SN-38 metabolism, which can lead to serious adverse drug reactions such as neutropenia. Therefore, conducting UGT1A1 genetic testing before administering IRI is recommended, particularly for patients with high serum bilirubin levels, the elderly, individuals in poor general health, and those who have previously experienced severe toxicity with IRI. However, predicting IRI toxicity based solely on genetic polymorphism should be approached with caution due to lack of reproducibility. Therefore, close monitoring and careful management of adverse reactions are essential during treatment, regardless of genetic testing results (44).

#### UGT1A1 polymorphism in Chinese population

In a study involving 276 patients with advanced CRC receiving Irinotecan-based chemotherapy, genotypes of UGT1A1-6 and UGT1A1-28 were determined using PCR amplification and Sanger sequencing. The impact of these genetic variations on severe diarrhea and neutropenia was explored. The results indicated that the UGT1A1-6 and UGT1A1-28 variants were present in 35.5% and 21.0% of the patients, respectively. Severe diarrhea was not associated with the presence of these variants. However, both UGT1A1-6 and UGT1A1-28 variants were significantly linked to severe neutropenia. The study found no differences between severe toxicities and clinical response. Interestingly, Chinese patients displayed distinct frequencies of the UGT1A1-6 and UGT1A1-28 genotypes compared to Western populations. Both UGT1A1-6 and UGT1A1-28 variants were closely connected to Irinotecan-induced severe neutropenia (45).

A study involving 138 patients with mCRC who received treatment with Irinotecan and Fluorouracil investigated the UGT1A1-28 and UGT1A1-6 alleles. The findings revealed a unique distribution of UGT1A1 genotypes among Chinese patients, which could potentially explain the comparatively lower levels of toxicity observed in mCRC patients treated with Irinotecan and Fluorouracil (46). A total of 356 locally advanced rectal cancer patients from multiple centers in China were enrolled on a randomized phase III trial for neoadjuvant chemoradiation using Capecitabine and Irinotecan. This trial stratified patients based on their UGT1A1-1 and UGT1A1-28 genotypes. The results showed that the addition of Irinotecan, guided by the UGT1A1 genotype, to Capecitabine-based neoadjuvant chemoradiotherapy led to a significant increase in complete tumor response among Chinese patients (47).

#### UGT1A1 polymorphism in Taiwanese and Taiwan Chinese populations

In a case-control study conducted in Taiwan, which included 709 participants consisting of CRC patients and healthy individuals, the study revealed that the combined presence of the UGT1A7-3 variant and the UGT1A1 211 allele significantly increased the risk of metastasis in CRC patients (48). A total of 112 healthy Taiwanese Chinese individuals and 505 Taiwanese Chinese UGT1A1 carriers underwent genotyping and sequencing for UGT1A1 and UGT1A7. The findings indicated that the allele frequencies of the UGT1A7 gene in the Taiwanese Chinese population differ from those in Caucasian and Japanese populations. Furthermore, the presence of the nucleotide 211 variant in the UGT1A gene was strongly associated with the UGT1A7-3 variant (49).

#### UGT1A1 polymorphism in Korean population

In a clinical trial enrolling Korean patients with mCRC, various UGT1A1-28 and UGT1A1-6 genotypes were assessed with respect to the number of defective alleles present (none, one, or two). The study aimed to investigate the suitability of Irinotecan dosing in combination with a fixed dose of Capecitabine in a phase I dose-escalation trial. The findings revealed that tailoring Irinotecan dosages based on UGT1A1-28 and UGT1A1-6 genotypes is a feasible approach. Furthermore, it was observed that higher Irinotecan doses can be safely administered to patients with either none or one defective allele compared to those with two defective alleles (50).

#### UGT1A1 polymorphism in Dutch population

A multicenter phase III trial conducted by the Dutch Colorectal Cancer Group aimed to explore the connections between the UGT1A1-28 genotype and the following factors in 218 CRC patients treated with Irinotecan. Main study outcomes included response rates, occurrence of febrile neutropenia, and maintenance of dose intensity. Patients with the UGT1A1-28/28 genotype exhibited a higher likelihood of experiencing febrile neutropenia when undergoing Irinotecan treatment. However, they were still able to receive similar doses and complete the same number of treatment cycles as patients with other genotypes. Response rates were found to be comparable among different genotypes. For patients with the UGT1A1-1/1 genotype, there were no significant differences in terms of the effectiveness of the treatment when compared to patients with other UGT1A1 genotypes. The study further confirmed that the UGT1A1-28 genotype is associated with a higher risk of febrile neutropenia, but it did not result in a reduction of Irinotecan doses. However, reducing the initial dose may lead to a lower incidence of febrile neutropenia in these patients (51).

#### UGT1A1 polymorphism in Brazilian population

The analysis of 12 different variants were described in the context of 125 cases of gastric cancer (GC), 66 cases of colorectal cancer, and 475 individuals without cancer. The study revealed that among these 12 variants, UGT1A1, along with 11 other polymorphisms found in genes associated with functions related to inflammatory pathways, immune response, and cellular and genomic stability (such as *CASP8, CYP2E1, CYP19A1, IL1A, IL4, MDM2, NFKB1, PAR1, TP53, TYMS*, and *XRCC1*) play a role in the development of both colorectal and gastric cancers. The UGT1A1 gene is responsible for the detoxification and metabolic processing of various substances within the liver. The specific marker studied within this gene, denoted as rs8175347, exhibited four possible alleles: UGT1A1-36 (5 repeats), UGT1A1-1 (6 repeats), UGT1A1-28 (7 repeats), and UGT1A1-37 (8 repeats). Among these, UGT1A1-1 was regarded as the wild-type allele and most observed allele, followed by the UGT1A1-28. The alleles UGT1A1-36 and UGT1A1-37 were considered rare. The study suggested that carrying at least one of the rare alleles in this polymorphism increases the risk of developing CRC thirteen-fold (52).

#### UGT1A1 polymorphism in Caucasian populations

A meta-analysis conducted in a Caucasian population was aimed at investigating the connection between UGT1A1-28 polymorphisms and the clinical outcomes of Irinotecan-based chemotherapies. The study suggested that UGT1A1-28 polymorphism could not be relied upon as a consistent predictor of both tumor response and progression-free survival (PFS) in CRC patients undergoing Irinotecan-based chemotherapy. The relationship between UGT1A1-28 and overall survival (OS) in patients treated with lower-dose Irinotecan chemotherapy was inconclusive and needed further validation. When analyzing data across various tumor types (including CRC), no significant association was observed between UGT1A1-28 genotypes and the response rate (53).

In a study involving 100 healthy Caucasians people and 50 Egyptians, researchers investigated the co-occurrence of a TATA box mutation associated with Gilbert’s syndrome (UGT1A1-28) along with other polymorphisms in the UDP-glucuronosyltransferase-1 locus (UGT1A1-6 and UGT1A1-73). The study found that allele frequencies did not significantly differ between the two populations, although Egyptians tended to have higher UGT1A1-71 allele frequency and lower UGT1A1-72 allele frequency. The remaining polymorphic alleles were consistent with Hardy-Weinberg equilibrium, suggesting they developed independently of evolutionary pressures. Haplotype frequencies were estimated to determine the co-occurrence of polymorphic variants of three UGT1 isoforms on the same chromosome. Interestingly, the study revealed that individuals homozygous for the UGT1A1-28 promoter mutation were also homozygous for the UGT1A1-6 and UGT1A1-73 allelic variants in both populations (54). Three main haplotypes were identified, with one containing allelic variants of UGT1A1-28, UGT1A1-6, and UGT1A1-72/3. This haplotype, found in 29% of Caucasians and 22% of Egyptians, was linked to decreased UGT activity (54).

#### UGT1A1 polymorphism in Israeli population

A review of 329 CRC cases diagnosed in Israeli revealed a strong association between the UGT1A1-28/28 genotype and severe hematologic toxicity, increased hospitalization rate, and decreased survival rate in CRC patients undergoing Irinotecan treatment (55).

### UGT1A1 polymorphism in other populations

In a literature review conducted between 1984 and 2013, global variations in the UGT1A1 gene were analyzed in a sample of 146 Lebanese without cancer and compared to other populations. This analysis revealed effects associated with different geographic distributions. Variations in the UGT1A1-28 gene resulted from a combination of interethnic disparities and geographical spread. Less efficient UGT1A1-28 variants were widespread in African and Southern Asian populations, whereas more active forms were prevalent in Eastern and Southeastern Asian populations (56). An investigation of anonymous DNA samples and ethnic variation within the UGT1A1 promoter emphasized its function as a balanced polymorphism that efficiently controls bilirubin metabolism. The study also analyzed the genotypes of individuals with Asian, African, and Caucasian heritage containing eight, seven, six, and five repeats on DNA samples. The study suggested that evolutionary forces may have countered somewhat unspecified genetic and environmental influences in determining the required number of repeats to maintain serum bilirubin levels in an ideal range. Even with changes in the number of promoter repeats, the continued existence of variations in bilirubin levels among different racial groups indicates that alterations in bilirubin metabolism likely occurred relatively recently, preventing complete adaptation to these changes (57).

A study involving 300 healthy Iranian individuals found the G/G genotype of UGT1A1-6 to be the most prevalent across various ethnicities within the Iranian population (58). Another study on 306 healthy volunteers from the three main ethnic groups in Malaysia (Malays, Chinese, and Indians) suggested the necessity of genotyping UGT1A1-6, UGT1A1-28, and UGT1A1-27 before prescribing Irinotecan. The study observed a higher frequency of the homozygous UGT1A1-28 (7TA/7TA) genotype in Malaysians and Indians compared to the Chinese, with no significant differences in the distribution of UGT1A1-6 and UGT1A1-27 among the groups. The UGT1A1-27 allele, not detected in Caucasian and African American populations, was found in Malaysians and Chinese people (59) (Table 1).

**TABLE 1:** UGT1A1 polymorphisms and their impact on CRC treatment across different populations.

Study name (identifier)	Year of publication	GI cancer subtype	Population type	Number of cases and controls included	UGT1A genotype studied	Treatment given to cases
Zhu X et al.	2020	Colorectal Cancer (CRC)	Mixed Population	1652 patients	UGT1A1-6	Irinotecan
Cecchin E et al.	2009	Metastatic CRC (mCRC)	Mixed Population	71 patients	UGT1A1-28, −60, −93, −73, −9–22	FOLFIRI
Shirasu et al.	2019	Pancreatic Cancer	Japanese patients with unresectable cancer	199 patients	UGT1A1-6, UGT1A1-28	FOLFIRINOX (standard and modified)
Gao et al.	2013	Advanced CRC	Chinese CRC patients	276 patients	UGT1A1-6, UGT1A1-28	Irinotecan-based chemotherapy
Tang et al.	2005	Colorectal Cancer	Taiwanese CRC patients	709 participants: 268 + 441 Healthy Controls	UGT1A7-3, UGT1A1 211	Surgery
Kweekel DM et al.	2008	Colorectal Cancer (CRC)	Dutch CRC patients	218 patients	UGT1A1-28	Irinotecan
Cavalcante et al.	2017	Colorectal and Gastric Cancer (GC)	Brazilian CRC and GC patients	666: 125 CRC, 66 GC patients + 475 cancer-free individuals)	UGT1A1 (rs8175347)	N/A*
Liu X et al.	2013	Colorectal Cancer (CRC)	Caucasian CRC patients	1444 patients (551 homozygous + 893 heterozygous)	UGT1A1-28	Irinotecan

*N/A: no applicable treatment.

### Pharmacogenomic: how different genomic or genetic characteristics affect drug metabolism in colorectal cancer

A meta-analysis showed that patients with the UGT1A1-28 allele(s) are at an increased risk of severe diarrhea induced by Irinotecan, especially at medium or high doses (56). A retrospective analysis of 173 mCRC patients treated with cetuximab or bevacizumab in combination with FOLFIRI categorized patients based on their UGT1A1 genotype. The study indicated the potential safety of higher Irinotecan doses for individuals with UGT1A1 6TA/6TA and UGT1A1 6TA/7TA genotypes, suggesting that dose adjustment based on pretherapeutic genotyping could improve outcomes (57). The ideal dosage for individuals with the UGT1A1-28 homozygous genetic variant remains uncertain. While a 20% dose reduction is suggested, dose escalation to full levels in subsequent treatment cycles may be considered if minimal or no toxicity is observed at the reduced dosage (58, 59).

UGT1A1 genotyping in the second-line treatment of colorectal cancer with high-dose Irinotecan administered once every 3 weeks, along with adjusting the initial Irinotecan dose for patients with the UGT1A1-7/7 genotype, resulted in cost savings for African and Caucasian populations. However, this genotyping approach was not cost-effective for an Asian population. Additionally, the proactive use of G-CSF in UGT1A1-7/7 genotype patients did not prove to be cost-effective across any population group. Finally, implementing a treatment strategy with a high dose every 3 weeks with a 20% reduction in dosage compared to the low-dose weekly Irinotecan regimen for patients with the *UGT1A1*7/7 genotype was not only more economical but also more convenient for the patients (60). Another retrospective study involving 105 mCRC patients treated with low-dose Irinotecan -based chemotherapy found that UGT1A1 gene polymorphism in the promoter region did not significantly affect treatment effectiveness or the occurrence of side effects (61). In 2005, the U.S. Food and Drug Administration (FDA) recommended the inclusion of UGT1A1-28 in patient labels to inform about potential drug metabolism variations. This proposal aimed to enhance personalized treatment plans and mitigate adverse drug reactions (62).

## Future directions and conclusion

Larger studies are needed to confirm these findings and develop personalized measures to predict Irinotecan-related toxicities. Such pharmacogenomic interventional clinical trials have struggled to gain traction due to funding. Foundation and governmental support are urgently needed to address this significant shortcoming in improving the safety profile and survival outcomes associated with current cancer care. Furthermore, the small sample sizes in current studies limit their generalizability (34), and national trials are crucial in this regard. While safety is crucial in the palliative setting, concerns about the necessity of dose modifications only for a specific patient group with UGT1A1-28/28 genotype needs to be addressed. Resources should be allocated to a comprehensive analysis of the different genotypes, including various UGT1A1 haplotypes and other genetic factors influencing treatment outcomes. There is an urgent need to establish appropriate dose modification strategies for Irinotecan-containing treatments across different populations and genotypes. Incorporating molecular studies to fine tune pharmacogenomics in both tumor and normal tissues may expand the goal of personalized therapy (59).

Data from proficiency testing programs to assess the analytical validity of clinical UGT1A1 tests, and more studies are needed to determine the clinical validity of tests for less common UGT1A1 variants. These studies require larger sample sizes because these genotypes are rare. To determine the appropriate Irinotecan dosage for patients with specific UGT1A1 genotypes, well designed randomized pharmacogenomic trials comparing outcomes between different patient populations are needed (9, 57). Personalized treatment plans for patients with cancer, to identify those at the risk of side effects from certain drugs are crucial in modern oncology practice. Pharmacogenomics can also be used to identify patients who are likely to respond well to certain drugs (63, 64). Experimental gene therapy to deliver a UGT1A1 encoding vector to patients with low or absent UGT1A1 activity is fascinating as an approach at reducing their risk of side effects (65). UGT1A1 may play a role in the development of resistance to cancer drugs. By understanding how UGT1A1 contributes to cancer resistance, we can develop new strategies to overcome it (66). Finally, challenges regarding access and insurance coverage for genetic testing must be addressed.
